# Feasibility study of three‐dimensional dose prediction of esophageal cancer radiotherapy based on CGAN

**DOI:** 10.1002/acm2.70683

**Published:** 2026-07-07

**Authors:** Qichao Hu, Yanrui Li, Youyi Tao, Yanxia Wang, Xudong Kong

**Affiliations:** ^1^ Department of Radiation Oncology Donghai County People's Hospital affiliated to Kangda College of Nanjing Medical University Lianyungang Jiangsu China; ^2^ Medical Physics in Radiation Oncology Department of Radiation Oncology Affiliated Hospital of Jiangnan University Wuxi Jiangsu China

**Keywords:** esophageal cancer, generative adversarial networks, radiotherapy, three‐dimensional dose prediction

## Abstract

**Objective:**

To evaluate the feasibility of a Conditional Generative Adversarial Network (CGAN)‐based model (2UDCNN‐AT‐GAN) for predicting 3D radiotherapy dose distributions in esophageal cancer, and to assess its potential utility as a guidance tool for Intensity‐Modulated Radiotherapy (IMRT) plan design.

**Methods:**

A CGAN‐based model was trained on 95 esophageal cancer IMRT plans and tested on 15 plans. The model integrated a dual‐path encoder‐decoder, attention‐gated fusion, and Dose‐volume histogram (DVH)‐based conditioning. Predicted dose distributions were compared with manual clinical plans using DVH metrics, plan evaluation parameters, and error analysis.

**Results:**

All predicted plans met clinical constraints (100% qualification rate). Predicted and manual plans identified concordant near‐limit organs at risk (OARs) cases. This consistency mirrored the critical OAR constraints that govern target dose distribution. The mean prediction error was 2.88 ± 2.88% with target errors of 1.00 ± 1.00%. The homogeneity index (0.08 ± 0.02) matched that of manual plans, while the conformity index (CI) was slightly lower (*p* = 0.0034). The mean Dice Similarity Coefficient (DSC) was 0.916.

**Conclusion:**

In this feasibility study, the 2UDCNN‐AT‐GAN model generated preliminary clinically acceptable dose predictions that met basic clinical constraints and showed spatial similarity to manual plans. A systematic ablation study confirmed that the integrated dual‐path and attention‐gated architecture outperforms individual components, establishing proof‐of‐concept feasibility for esophageal IMRT planning guidance. However, systematic target underdosage and the single‐institution scope limit its current role to a preliminary planning reference.

## 1 INTRODUCTION

Esophageal cancer is a malignancy with high incidence and mortality rates in China.^1^ Patients are frequently diagnosed at advanced stages, necessitating large radiotherapy target volumes. Intensity‐modulated radiotherapy (IMRT) planning is challenging due to irregular and elongated target morphology, dense distribution of radiosensitive organs at risk (OARs) around the esophagus, and stringent OAR dose constraints. These factors make IMRT planning operator‐dependent.

Radiotherapy dose prediction methods, including traditional knowledge‑based planning (KBP) and deep learning, fall under generalized knowledge‑based planning (GKBP). GKBP learns from high‑quality plan datasets to guide new plan design, with three key components: learning objects, methods, and objectives.

Learning targets range from manually extracted features (e.g., Overlap Volume Histogram (OVH),^2^ Distance Target Histogram (DTH)^3^, ^4^) to complete data (Computed Tomography (CT) images, contours) containing high‐dimensional feature information. Learning methods include machine learning (generalized principal component analysis (gPCA)^3^, ^5^) and deep learning architectures including convolutional neural networks (CNNs) such as U‐Net,^6^ residual U‐Net,^7^ Generative Adversarial Network (GAN),^8^, ^9^ often combined with attention mechanisms.^9^ The learning objective is typically DVH parameters or 3D dose distributions.

For esophageal cancer, GKBP studies using manual features (DTH,^10^, ^11^ multi‐regions of interest (ROI) features^12^) suffer from restricted informational scope,^13^ omission of critical features,^14^ and high time consumption. Hence, CT images or contours are used,^15^ despite redundancy and extraction challenges. Recent studies have applied U‐Net^16^ or residual U‐Net^17^ for dose prediction.

Esophageal IMRT faces unique challenges: the Planning Target Volume (PTV) is elongated and irregular with variable OAR proximity, and beams produce broad low‑dose distributions affecting the lungs. A dual‑path encoder‑decoder captures global anatomy and local target morphology, while attention‑gated fusion enhances PTV‑OAR boundaries where dose gradients are steepest. Their integration is hypothesized to improve spatial dose fidelity and OAR sparing. We thus proposed a Conditional Generative Adversarial Network (CGAN)^18^ framework integrating a dual‑path U‐Net,^19^ attention‐gated^9^ fusion, and DVH‑based conditioning—a combination not systematically evaluated for esophageal cancer. This pilot feasibility study assesses clinically acceptable dose prediction, spatial similarity to manual plans, and limitations, aiming to establish technical viability as a preliminary planning reference tool, not clinical equivalence.

## 2 MATERIALS AND METHODS

### 2.1 Data collection and processing

We retrospectively analyzed 110 esophageal cancer patients (86 males, median age of 74 years) treated with IMRT (predominantly 5‐field, prescribed as 50 Gy in 25 fractions to cover the PTV D_95%_). A stratified random test set of 15 cases was selected. Data processing included: (1) CT standardization and 3D reconstruction; (2) extraction of PTV and OARs (lungs, heart, spinal cord), along with voxel labeling and DVH parameters as listed in Table 1; (3) dose resampling and registration; and (4) extraction of multiple contiguous slices (± 4 slices centered on the PTV) for training.

**TABLE 1:** Structure labeling and dosimetric parameters.

Structure name	Patient	Lungs	Heart	Spinal Cord	PTV
Label value	10	13	26	45	47
DVH value	None	$\;D_{mean},V_{20},V_{5}$	$D_{mean},V_{30},V_{40}$	$\;D_{max}$	$\;D_{95\%},D_{98\%},D_{50\%},D_{2\%}$

### 2.2 Model development

We proposed a CGAN‑based model named 2UDCNN‑AT‑GAN. The generator synthesized a 256×256 dose map from three inputs: CT images, structure contours, and PTV masks (using five consecutive slices). Normalized DVH parameters of OARs and PTV were added as conditional inputs. The discriminator classified real vs. synthetic dose maps with the same DVH constraints.

**FIGURE 1:**
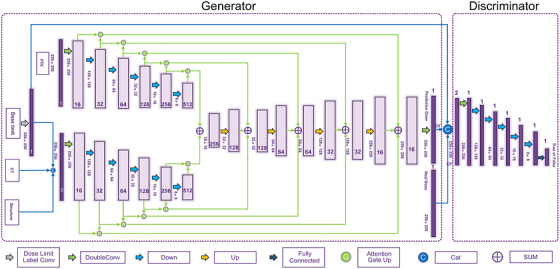
Neural network architecture of the 2UDCNN‐AT‐GAN model.

**FIGURE 2:**
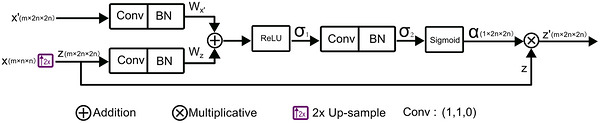
Implementation mechanism of the UP‐AG.

The generator used a dual‐path encoder‐decoder (Figure 1). One path processed PTV slices; the other processed CT and contours. The decoder replaced standard skip connections with attention‐gated up‐sampling modules (UP‐AG, Figure 2), which computed attention weights to fuse encoder and decoder features through weighted summation, thereby prioritizing relevant regions and suppressing noise.

The objective function, defined by Equation (1), combined adversarial loss and L1 loss:

(1) $$\begin{array}{c} min_{G}max_{D}L\left(D,G\right)=E_{x∼p_{data}\left(x|y\right)}\;\left[\log\left(D\left(x|y\right)\right)\right]\\ +E_{z∼p_{z}\left(z|y\right)}\left[\log\left(1-D\left(G\left(z|y\right)\right)\right)\right]\\ +\lambda E_{x∼p_{data}\left(x|y\right),z∼p_{z}\left(z|y\right)}\left[x-G{\left(z|y\right)}_{1}\right]\# \end{array}$$

where x is the real dose, z is the input (CT, structures, PTV), y are the DVH constraints, and λ=90 balances the L1 penalty.

The model was trained using PyTorch, Adam optimizer (G: 2e‐4, D: 3e‐4), batch size 32. See Table 2 for full configuration.

**TABLE 2:** Model training configuration and parameters.

Component	Parameter	Specification
Core modules	DoubleConv	[Conv (k, s, p: 3,1,1), BatchNorm, LeakyReLU] ×2
	Down	[MaxPool (k, s, p: 3,2,1), DoubleConv, ResidualBlock]
	UP	[UpSample(scale_factor: 2), DoubleConv, UP‐AG]
Pathway channels and size: c(s)^a^	Generator	Encoder: 8(256) →16(256) →32(128) →64(64) →128(32) →256(16) →512(8) Decoder: 512(8) →256(16) →128(32) →64(64) →32(128) →16(256) →1(256)
	Discriminator	2(256) →1(256) →1(128) →1(64) →1(32) →1(16) →1(8) →64(1) →32(1) →16(1) →1(1)
Training setup	Optimizer (lr)	Adam (G: 2e‐4, D: 3e‐4)
	Loss function	BCE
	Batch size	32
	λ (L1 penalty)	90

^a^ C: channels; S: spatial size (height × width).

### 2.3 Plan evaluation and model performance metrics

Generated plans were evaluated against clinical constraints (OAR doses), and PTV dose compliance was graded: A (50 Gy ± 1%), B (± 2%), C (± 5%), D (> 5%). Conformity Index: $CI=\frac{V_{R\cap PTV}}{V_{PTV}}$, where $V_{R\cap PTV}$ is the intersection of an isodose surface with the PTV, and $V_{PTV}$ is the target volume.

Homogeneity Index: $HI=\frac{D_{2\%}-D_{98\%}}{D_{50\%}}$, where $D_{x\%}$ denotes the dose of x% PTV volume. DVH parameters are listed in Table 1. The Dice Similarity Coefficient (DSC) quantifies the spatial overlap between predicted and manual isodose surfaces: $DSC=\frac{2|A\cap B|}{\left|A|+|B\right|}$, where A and B are binary masks of the two regions.

### 2.4 Statistical analysis

All statistical analyses were performed using SPSS 23.0. DVH‐based parameters were compared between predicted and manual plans using paired tests. Normality of differences was assessed with the Shapiro–Wilk test. Data are mean ± SD or median (IQR) for normal and non‐normal distributions, respectively. Normally distributed data were analyzed with paired *t*‐tests, while non‐normal data were analyzed with Wilcoxon signed‐rank tests. Bonferroni correction was applied for multiple comparisons across eight parameters (significance threshold: α=0.0068). The percentage difference for each DVH parameter was calculated using Equation 2, where $DVH_{prediction}$ and $DVH_{real}$ represent the predicted and clinical manual plan values, and $DVH_{prescription}$ is the prescribed dose.

(2) $$DVH_{\Delta }=\frac{\left|DVH_{prediction}-DVH_{real}\right|}{DVH_{prescription}}\;\times 100$$

## 3 RESULTS

All predicted plans met clinical dose constraints (100% pass rate). Quantitative comparisons between predicted and manual plans were summarized in Tables 3 and 4. Predicted plans showed systematic differences from manual plans. The mean PTV D_95%_ was 49.23 ± 0.71 Gy (manual: 49.79 ± 0.27 Gy), and only 6 of 15 predicted plans achieved a “Plan A” rating compared to the vast majority of manual plans. The CI was significantly lower in predicted plans (p=0.0034), while the HI was comparable (0.08 ± 0.02 vs. 0.08 ± 0.01). The mean prediction error across all DVH parameters was 2.88 ± 2.88%, with the target error lower (1.00 ± 1.00%) than that of OARs.

**TABLE 3:** Dosimetric parameter comparisons between predicted and manual plans: Differences and statistical significance (*p*‐values).

	Clinical indices	Predicted plan	Manual plan	*p*‐value	Difference	Overall average error
PTV 𝐷_95%_	50 Gy	49.23 ± 0.71 Gy	49.79 ± 0.27 Gy	0.0089	1.00 ± 1.00%	2.88 ± 2.88%
Lungs 𝐷_mean_	≤13 Gy	7.24 ± 2.60 Gy	7.53 ± 2.70 Gy	0.0246	3.00 ± 2.00%	
Lungs 𝑉_20_	≤25%	12.55 ± 6.32%	13.26 ± 6.35%	0.2816	8.00 ± 7.00%	
Lungs 𝑉_5_	≤60%	39.34 ± 3.93%	38.86 ± 3.17%	0.3536	2.00 ± 2.00%	
Heart 𝐷_𝑚𝑒𝑎n_	≤26 Gy	1.22(0.09,8.19)	1.22(0.09,7.05)	0.0630	1.00 ± 1.00%	
Heart 𝑉_30_	≤40%	0.00(0.00,8.59)	0.00(0.00,6.75)	0.1282	2.00 ± 3.00%	
Heart 𝑉_40_	≤30%	0.00(0.00,3.52)	0.00(0.00,2.65)	0.0425	1.00 ± 2.00%	
Spinal Cord 𝐷_max_	≤50 Gy	41.25 ± 6.88 Gy	41.32 ± 8.16 Gy	0.9468	6.00 ± 5.00%	
HI		0.08 ± 0.02	0.08 ± 0.01	0.3560	/	
CI		0.90(0.80,0.94)	0.95(0.94,0.96)	0.0034		

**TABLE 4:** Comparative distribution of predicted vs. manual plans across four quality grades.

	Plan grading assessment			
	A (50 Gy ± 1%)	B (50 Gy ± 2%)	C (50 Gy ± 5%)	*D* (≥5%)
Manual plan	14	1	0	0
Predicted plan	6	4	5	0

Table 5 presents the model's predictions for representative boundary cases selected from the 15 test plans (the case with the minimum PTV D_95%_ and those where OAR doses were closest to their respective clinical constraints). The near‐limit OAR cases in the model predictions were concordant with those in the manual plans.

**TABLE 5:** Boundary cases: Predicted vs. manual plans with minimum PTV D95% and near‐limit OAR doses.

	Cases	PTV	Lungs			Heart			Spinal cord
		𝐷_95%_	𝑉_20_	𝑉_5_	𝐷_mean_	𝑉_40_	𝑉_30_	𝐷_mean_	𝐷_max_
Predicted plan	Case 2	49.33	20.41	53.04	10.43	9.22	3.35	5.88	48.66
	Case 8	47.78	7.87	30.79	5.17	0.00	0.00	0.00	42.10
	Case 9	50.31	7.43	35.08	6.67	11.45	5.28	16.19	36.03
	Case 15	49.29	23.28	55.66	11.07	0.00	0.00	0.00	43.94
Manual plan	Case 2	49.77	22.00	51.85	11.04	6.49	1.66	5.81	49.77
	Case 9	50.52	14.23	38.29	7.70	12.22	5.44	16.71	34.10
	Case 15	49.74	22.1	50.37	10.57	0.00	0.00	1.22	46.39

**FIGURE 3:**
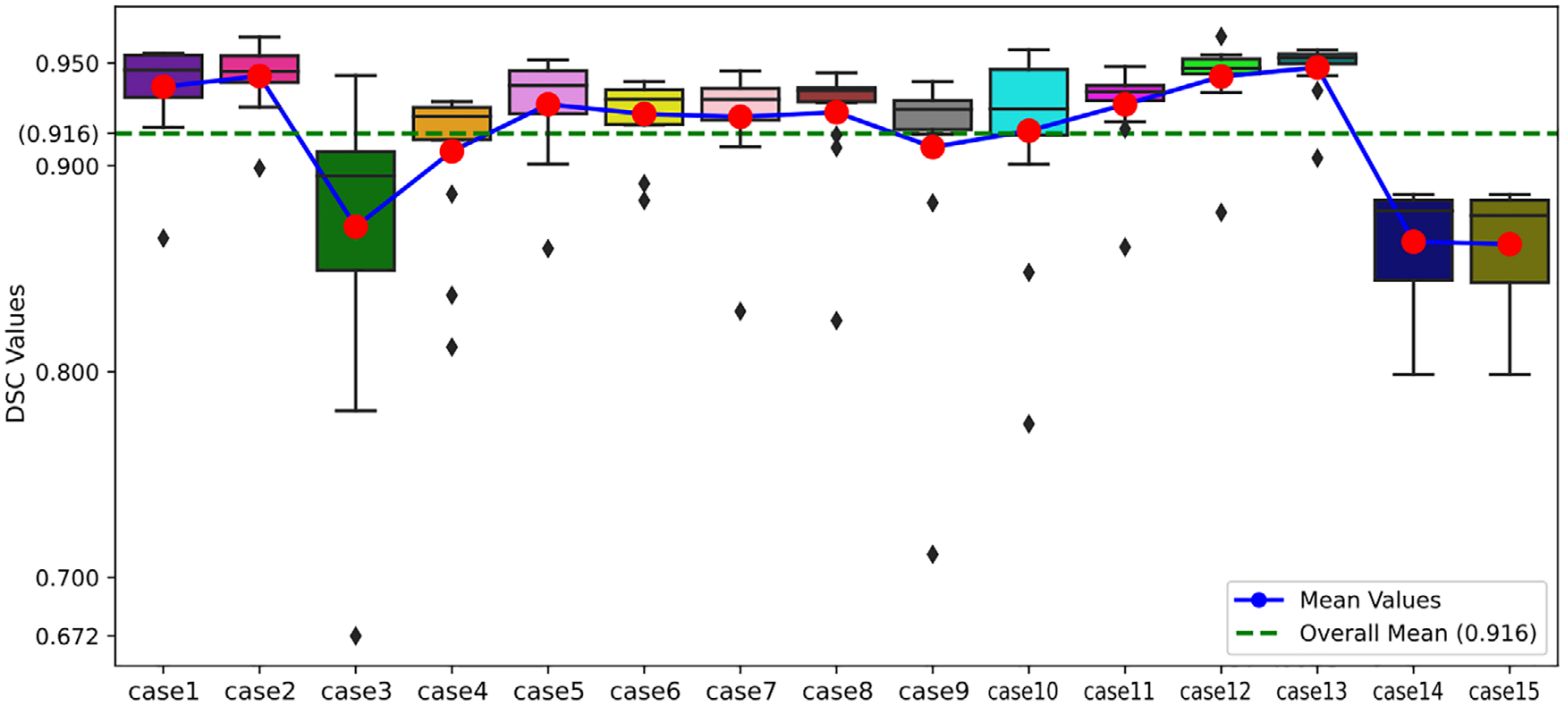
DSC comparison of isodose surfaces between automated and manual radiotherapy plans (14 dose gradients across 15 cases).

**FIGURE 4:**
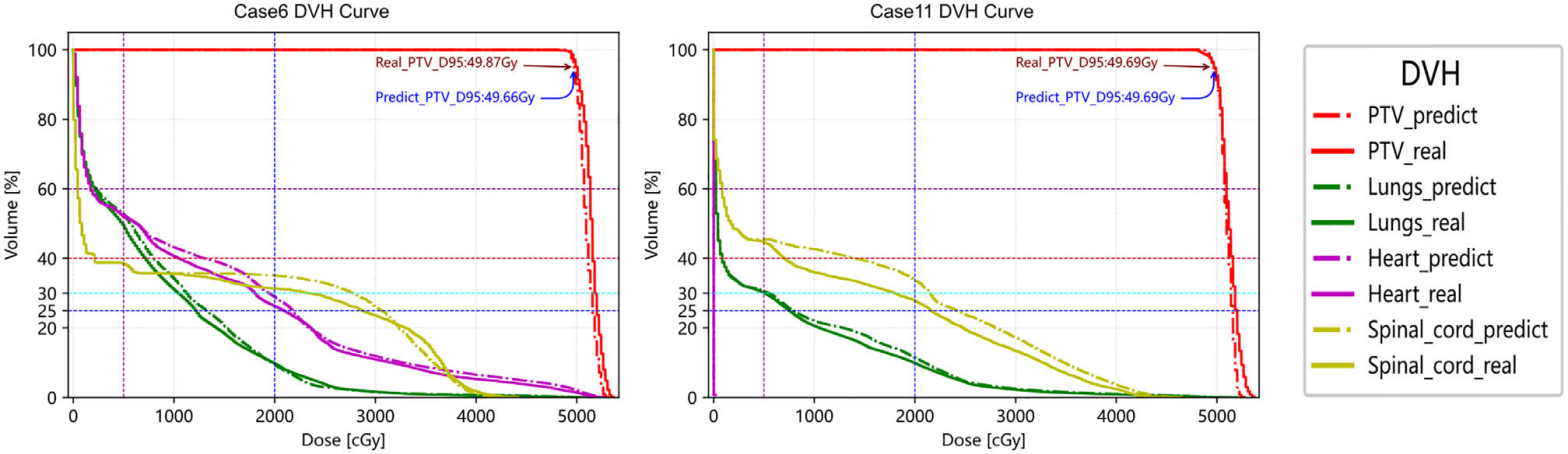
DVH curve comparison between predicted and manual plans for two A‐graded cases.

**FIGURE 5:**
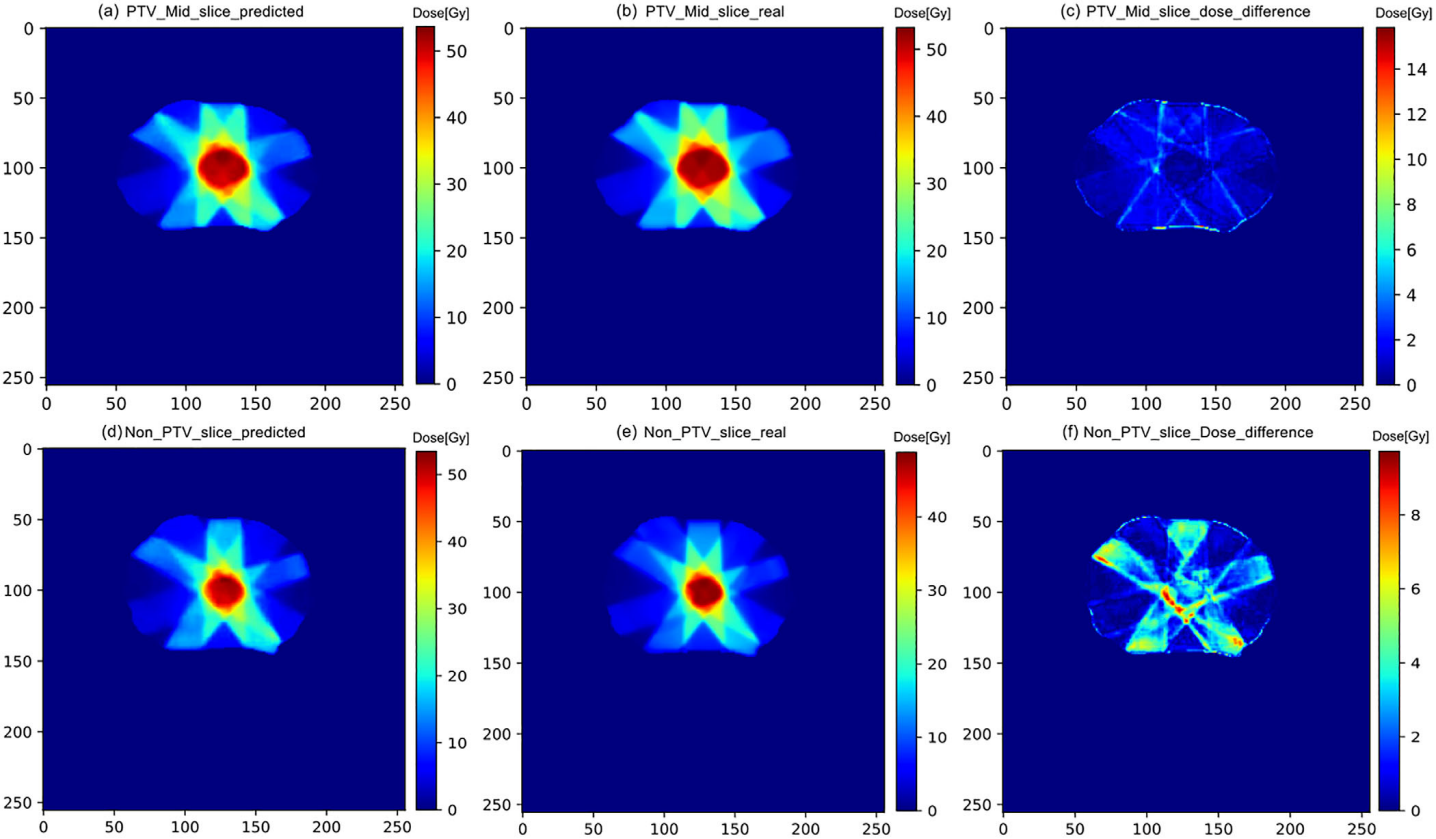
Case 6: Dose distribution in the mid‐PTV slice and non‐PTV slice.

Spatial similarity, measured by the DSC across 14 isodose levels (15–50 Gy), produced a mean DSC of 0.916 (Figure 3). Representative case analyses are shown in Figures 4 and 5. Figure 4 presents DVH curves for two “A”‐graded predicted plans (Cases 6 and 11) alongside their manual plans. Key constraints (e.g., lung $V_{5}\leq 60\%$, $V_{20}\leq 25\%$) and PTV D_95%_ are marked. Solid and dashed lines represent manual and predicted plans, respectively; structures are color coded (PTV in red, spinal cord in yellow, heart in purple, lung in green). Figure 5 compares predicted and manual dose distributions for Case 6 at a mid‐PTV slice (first row) and a non‐PTV slice (second row), including absolute difference maps (calculated as $Dose_{\Delta }=\left|Dose_{prediction}-Dose_{real}\right|$). Table 6 quantifies the pixel‐wise Mean Absolute Error (MAE) within the patient contour for both slices in Cases 6 and 11.

**TABLE 6:** Dose distribution discrepancy analysis: Predicted vs. manual plans in mid‐PTV and non‐PTV slices (pixel‐wise MAE within the patient contour, case 6 & 11).

Case	mid‐PTV slice (Gy)	non‐PTV slice (Gy)
Case 6	1.29	1.99
Case 11	0.7	2.94

In an ablation study under identical training conditions, we compared four variants: Baseline, Dual, AG, and AG‑Dual (Table 7). The full AG‑Dual model achieved the lowest MAE (0.0439) and root mean square error (RMSE: 0.1123), lungs 𝑉_20_ error (24.14%), heart 𝐷_𝑚𝑒𝑎n_ error (7.30%), and spinal cord 𝐷_max_ error (13.45%), as well as the highest mean DSC (0.7622) and DSC@40 Gy (0.8534). Although the AG variant showed slightly lower PTV 𝐷_95%_/𝐷_98%_ errors, AG‑Dual provided substantially better OAR protection and spatial accuracy, confirming complementary benefits between the two components.

**TABLE 7:** Ablation study results comparing four model variants.

			Error (%)						
Model	MAE	RMSE	PTV 𝐷_95%_	PTV 𝐷_98%_	Lung 𝑉_20_	Heart 𝐷_mean_	SC 𝐷_max_	Mean DSC	DSC@40 Gy
Baseline	0.0492	0.1285	8.20	10.21	64.57	14.03	25.56	0.6380	0.6381
Dual	0.0496	0.1353	5.79	7.62	31.53	16.45	15.09	0.7498	0.8181
AG	0.0528	0.1711	4.21	4.71	45.12	11.14	18.53	0.7119	0.7144
AG‐Dual	0.0439	0.1123	5.37	7.88	24.14	7.30	13.45	0.7622	0.8534

*Note*: Bold values indicate optimal performance. $Error\left(\%\right)=\frac{|DVH_{pred}-DVH_{real}|}{DVH_{real}}\;\times 100$. DSC@40 Gy denotes the DSC at the 40 Gy isodose surface. SC denotes the Spinal Cord. Individual per patient data are available in Table S1.

## 4 DISCUSSION

This study evaluated a CGAN‑based model for predicting 3D dose distributions in esophageal cancer IMRT, assessing its feasibility as a planning reference (not an autonomous plan generator).

The model demonstrated several promising characteristics. All predicted OAR doses satisfied clinical constraints (100% pass rate, Table 4). The mean prediction error was 2.88% ± 2.88% overall, with PTV errors lower (1.00% ± 1.00%, Table 3). The mean DSC across 14 isodose levels was 0.916, indicating moderate‑to‑high spatial similarity to manual plans (Figure 3). Beyond basic acceptability, the model and manual plans identified the same near‐limit OAR cases that most constrained PTV coverage.

Ablation results (Table 7) revealed complementary roles of the two modules. Dual alone improved spatial dose fidelity (DSC@40 Gy +28.2%) and reduced lungs/spinal cord errors, but increased MAE/RMSE slightly. AG alone excelled at target coverage (PTV 𝐷_95%_/𝐷_98%_ errors reduced by ∼50%) and heart 𝐷_mean_ error, yet RMSE rose by 33.2%. The integrated AG‐Dual model achieved the best balance: lowest MAE/RMSE, highest DSC@40 Gy (0.8536), and optimal OAR sparing. The dual‑path stabilizes global dose distribution; AG sharpens critical boundaries. This confirms our hypothesis that both modules address distinct challenges of esophageal IMRT—their synergy yields superior overall performance.

Based on these capabilities, the model's predictions offer practical guidance for IMRT plan design in three main ways. First, the predicted DVH values (e.g., lungs $V_{5}$, $V_{20}$, spinal cord

$\;D_{max}$) can be used as initial optimization constraints, providing a reasonable starting point for inverse planning. Second, by pinpointing the dose‐limiting factors (“bottlenecks”) that limit target dose delivery—i.e., identifying which OAR most constrains the PTV—planners can prioritize that OAR during optimization (e.g., by assigning higher weighting factors, adjusting beam directions, or relaxing less critical objectives). Third, the predictions help assess the potential for target dose escalation. When predicted OAR doses are well below their tolerance limits, there is considerable room to increase PTV dose without violating constraints; for example, in Case 8 (Table 5), low predicted lung and heart doses suggest a possibility for dose escalation.

These characteristics indicate that the model could provide initial, evidence‑based guidance for setting planning objectives. However, the observed systematic underdosage currently limits its use to hypothesis generation rather than direct plan acceptance.

Major limitations: (1) systematic target underdosage (only 40% achieved grade A, lower CI, p=0.0034; PTV D_95%_ difference did not reach significance after Bonferroni correction, p=0.0089>0.0068). This reflects that the absolute prediction errors remain above clinically ideal thresholds, despite the architectural improvements demonstrated in the ablation study (Table 7); (2) single‐institution test set (*n* = 15) limits statistical power and generalizability.

Additionally, larger OAR errors occurred in non‐PTV regions with steep dose gradients (Figure 5; Table 6). These errors are likely due to field‐edge dose fall‐off, anatomical variation, and the slice‐based approach lacking full 3D correlations. Limited training data and missing beam geometry may also contribute to these discrepancies.

Future work will focus on fully 3D models using multi‐institutional datasets, corrections for target underdosage (e.g., modified loss functions), and direct comparisons with state‐of‐the‐art methods (e.g., DoseGAN). Hence, this study is a proof‐of‐concept feasibility assessment: the model provides preliminary dose estimates for planning reference, not clinical equivalence.

## 5 CONCLUSION

In this feasibility study, the 2UDCNN‑AT‑GAN model generated preliminary dose predictions that met basic clinical constraints and showed moderate‑to‑high spatial similarity to manual plans. However, while an ablation study demonstrated the complementary benefits of the dual‐path and AG components, systematic target underdosage and the limited test set size restricts its current role to a proof‑of‑concept planning reference. Future work will focus on multi‐institutional validation, fully 3D architectures, and target coverage corrections.

## SUPPLEMENTARY DATA

The file Supplementary Table S1.xlsx contains per patient pixel‐wise errors (normalized dose domain) and Dice similarity coefficients for all four models. The README sheet explains all abbreviations and the normalization range.

## AUTHOR CONTRIBUTIONS

**Qichao Hu**: Methodology, investigation, writing—original draft, validation, software. **Xudong Kong**: Supervision, project administration. **Yanrui Li**: Resources, formal analysis, data curation, validation, writing—review and editing. **Youyi Tao**: Conceptualization, investigation, formal analysis, visualization, writing—review and editing. **Qichao Hu**, **Yanrui Li** and **Youyi Tao**: Contributed equally to this work. **Yanxia Wang**: Resources, data curation, validation.

## CONFLICT OF INTEREST STATEMENT

The authors declare no conflicts of interest.

## ETHICAL APPROVAL

This retrospective study was reviewed and approved by the Medical Ethics Committee of Donghai County People's Hospital (approval No. 2025‐KY‐008, dated May 30, 2025). The requirement for informed consent was waived by the same committee under Article 39 of the Ethical Review Measures for Biomedical Research Involving Human Subjects (《涉及人的生物医学研究伦理审查办法》, National Health Commission of China), as the study involved analysis of fully de‐identified hospital records with no direct patient contact, experimental interventions, or collection of identifiable personal information. All direct identifiers (e.g., names, ID numbers, admission dates) were removed prior to analysis, and data handling followed institutional privacy protection protocols to prevent re‐identification.

## Supporting information




Supporting Information

Supporting Information

## Data Availability

The data that support the findings of this study are available within the article. Raw patient data are not publicly available due to privacy restrictions under applicable Chinese law and institutional policy. Requests for de‐identified data may be directed to the corresponding author.
