# Supplementary material for: Feasibility study of three‐dimensional dose prediction of esophageal cancer radiotherapy based on CGAN
Source: J Appl Clin Med Phys. 2026 Jul 7;27(7):e70683. doi: 10.1002/acm2.70683 (PMC13341968; doi:10.1002/acm2.70683)
Supplement: Supplementary file 1 — Supporting Information [file ACM2-27-e70683-s001.pdf]

东海县人民医院

医学伦理委员会审查批件

审批号：2025-KY-008

研究项目名称	基于生存对抗网络的食管癌放射治疗剂量预测		
牵头研究单位	东海县人民医院放疗科	主要研究者/项目负责人	胡岐超
协作单位	——	主要研究者/项目负责人	——

审核材料：

1. ☑伦理审查申请表
2. ☑科研项目申报书
3. ☑知情同意书
4. ☐免除知情同意审查表

伦理审查方式	会议审查
审查时间	2025 年 5 月 29 日
审查委员	东海县人民医院医学伦理委员会委员

医学伦理委员会审查意见：

1、经伦理委员会对项目审查，同意开展该项临床研究。

2、伦理委员会对该研究进行年度/定期跟踪审查，跟踪审查频率为 12 个月，自同意研究日起，按照跟踪审查频率交年度/定期跟踪审查报告，请在 2026 年 5 月 30 日到期前 1 月递交。

3、此伦理批件的有效期为 2025 年 5 月 30 日— 2026 年 5 月 30 日，逾期未实施的，本批件自动失效。

主任委员签字：[signature]

批复日期： 2025 年 5 月 30 日

东海县人民医院医学伦理委员会（盖章）

声明：

1、本伦理委员会的职责、人员组成和工作流程遵循 ICH GCP、中国 GCP，符合《赫尔辛基宣言》的原则，并遵守中国相关法律和法规的规定。

2、研究过程中若变更主要研究者，对临床研究方案、知情同意书、招募材料等的任何修改，请申请人提交修正案审查。

3、如果试验中研究者获知任何严重不良事件请于 24 小时内通知本伦理委员会。

4、重大违背或偏离方案应及时提交违背/偏离方案报告表。

5、申请人暂停或提前终止临床研究，请及时提交暂停/终止研究报告。

6、完成临床研究，请申请人提交结题报告。

联系地址：江苏省连云港市东海县富国路 299 号

联系电话：0518-80303222　　　　电子邮箱：dhywk2009@163.com
